# Association of Participation in the Maryland Primary Care Program With COVID-19 Outcomes Among Medicare Beneficiaries

**DOI:** 10.1001/jamanetworkopen.2022.49791

**Published:** 2023-01-06

**Authors:** Emily Gruber, Chad Perman, Rachel Grisham, Eli Y. Adashi, Howard Haft

**Affiliations:** 1 Maryland Primary Care Program, Maryland Department of Health, Baltimore; 2 Warren Alpert Medical School, Brown University, Providence, Rhode Island

## Abstract

**Importance:**

Advanced primary care is a team-based approach to providing higher-quality primary care. The association of advanced primary care and COVID-19 outcomes is unknown.

**Objective:**

To evaluate the association of advanced primary care with COVID-19 outcomes, including vaccination, case, hospitalization, and death rates during the first 2 years of the COVID-19 pandemic.

**Design, Setting, and Participants:**

This retrospective cohort study used Medicare claims data from January 1, 2020, through January 31, 2022, and Maryland state vaccination data. All Part A and B Medicare claims for Maryland Medicare beneficiaries were included. The study population was divided into beneficiaries attributed to Maryland Primary Care Program (MDPCP) practices and a matched cohort of beneficiaries not attributed to MDPCP practices but who met the eligibility criteria for study participation from January 1, 2020, through December 31, 2021. Eligibility criteria for both groups included fee-for-service Medicare beneficiaries who were eligible for attribution to the MDPCP. A forced-match design was used to match both groups in the study population by age category, sex, race and ethnicity, Medicare-Medicaid dual eligibility status, COVID-19 Vulnerability Index score, Maryland county of residence, and primary care practice participation.

**Exposures:**

Primary care practice participation in the MDPCP.

**Main Outcomes and Measures:**

Primary outcome variables included rate of vaccination, monoclonal antibody infusion uptake, and telehealth claims. Secondary outcomes included rates of COVID-19 diagnosis, COVID-19 inpatient claims, COVID-19 emergency department claims, COVID-19 deaths, and median COVID-19 inpatient admission length of stay. Claims measures were assessed from January 1, 2020, through October 31, 2021. Vaccination measures were assessed from January 1, 2020, through March 31, 2022.

**Results:**

After matching, a total of 208 146 beneficiaries in the MDPCP group and 37 203 beneficiaries in the non-MDPCP group were included in this study, comprising 60.10% women and 39.90% men with a median age of 76 (IQR, 71-82) years. Most participants (78.40% and 78.38%, respectively) were White. There were no significant demographic nor risk measure baseline differences between the 2 groups. The MDPCP beneficiaries had more favorable primary COVID-related outcomes than non-MDPCP beneficiaries: 84.47% of MDPCP beneficiaries were fully vaccinated, compared with 77.93% of nonparticipating beneficiaries (*P* < .001). COVID-19–positive beneficiaries in MDPCP also received monoclonal antibody treatment more often (8.45% vs 6.11%; *P* < .001) and received more care via telehealth (62.95% vs 54.53%; *P* < .001) compared with nonparticipating counterparts. In terms of secondary outcomes, beneficiaries in the MDPCP had lower rates of COVID-19 cases (6.55% vs 7.09%; *P* < .001), lower rates of COVID-19 inpatient admissions (1.81% vs 2.06%; *P* = .001), and lower rates of death due to COVID-19 (0.56% vs 0.77%; *P* < .001) compared with nonparticipating beneficiaries.

**Conclusions and Relevance:**

These findings suggest that participation in the MDPCP was associated with lower COVID-19 case, hospitalization, and death rates, and advanced primary care and COVID-19 response strategies within the MDPCP were associated with improved COVID-19 outcomes for attributed beneficiaries.

## Key Points

**Question:**

Are the advanced primary care services provided by primary care practices in the Maryland Primary Care Program (MDPCP) associated with better COVID-19 outcomes, including vaccination, case, hospitalization, and death rates?

**Findings:**

In this cohort study of Medicare beneficiaries attributed to the MDPCP and matched with nonattributed Medicare beneficiaries, MDPCP beneficiaries had higher vaccination rates, monoclonal antibody therapy uptake, and use of telehealth services. In turn, MDPCP beneficiaries experienced lower COVID-19 case, hospitalization, and death rates.

**Meaning:**

These findings suggest that advanced primary care and COVID-19 response strategies within the MDPCP were associated with improved COVID-19 outcomes for attributed beneficiaries.

## Teaser

This cohort study evaluates COVID-19 outcomes among Medicare beneficiaries who participated in the Maryland Primary Care Program, an initiative that supported a team-based approach to improving quality of care in primary care practices.

## Introduction

The COVID-19 pandemic created major challenges for both the public health sector and primary care community in the US. For public health, challenges included providing clear public communication and messaging, allocating scarce resources effectively, ensuring high vaccination rates, and mobilizing an underfunded and understaffed workforce amidst the pandemic response.^1,2,3^ Primary care was challenged by a rapid transition to telehealth, the need to provide usual care on top of expanded pandemic-related services, high levels of stress and burnout, concerns of vaccine-hesitant patients, and a lack of resource provision from public health.^4,5,6^ Novel methods of primary care provided an opportunity for collaboration between public health and primary care to address overlapping challenges and create a strong, integrated approach.

The Maryland Primary Care Program (MDPCP) produced a model of integration between primary care and public health in the pandemic response.^7^ Created by the Maryland Department of Health (MDH) and the Centers for Medicare & Medicaid Services (CMS), the MDPCP enhances primary care by engaging over 500 practices directly with funding, data, technical assistance, and administrative resources.^7,8^ This engagement and relationship with MDH intends to transform care delivery to an advanced primary care paradigm, which expands the role of primary care to include services such as expanded care management, integrated behavioral health, data-informed care, and social needs screenings and referrals.

Enrollment in the MDPCP is voluntary, and primary care practices can apply to participate as a practice on an annual basis. The CMS reviews all applications for program eligibility criteria, which include attribution of at least 125 qualifying Medicare fee-for-service beneficiaries, appropriate clinician specialties, and use of certified electronic health record technology.^8^ When a practice participates in the MDPCP, they are assigned an MDH practice coach who facilitates the relationship between the practice and the state public health department. The MDPCP is unique in its integration of primary care and state public health.^9^

This article is a continuation of a 2021 Milbank Memorial Fund study^10^ that examined the association of public health and primary care partnership with COVID-19 outcomes among MDPCP beneficiaries in 2020 and found that MDPCP participation was associated with significantly lower rates of COVID-19 cases, hospitalizations, and deaths. This present study added the vaccination rate of the MDPCP-attributed population and rate at which beneficiaries received monoclonal antibody treatment, both of which are impacted by primary care and influence COVID-19–related outcomes.^11^ Additionally, the time horizon of the analysis was extended from January 1 through October 31, 2021.

The MDH used the existing infrastructure and relationships with primary care practices via the MDPCP to engage practices in the pandemic response. The MDPCP supported practices in the COVID-19 response in 4 key areas: (1) providing data tools to enable targeted outreach; (2) early and coordinated resource distribution, including vaccine allocation; (3) consistent communication between the MDH and practices; and (4) non–visit-based financial support for MDPCP practices.

The first coordination focus area was the deployment of data tools to primary care to better inform delivery of care. The MDH worked with the Chesapeake Regional Information System for Our Patients (CRISP), the Maryland state-designated health information exchange, to give practices access to up-to-date COVID-19 data for their beneficiaries through their program-supported dashboards and reports.^12^

A key resource that resulted from this partnership was a vaccine tracking system developed by CRISP that shows real-time vaccination data for beneficiaries.^13^ This system used data from ImmuNet, Maryland’s Immunization Information System, to display detailed vaccination status and key demographic and clinical data.^14^ When COVID-19 vaccine booster doses were recommended by the Centers for Disease Control and Prevention, the MDH worked with CRISP to rapidly create an “eligible for booster” filter within their tracking system so that practices could quickly identify who in their practice needed direct outreach to recommend a booster dose. Access to these data allowed practices to identify vulnerable patients and proactively monitor vaccine uptake among their beneficiaries.

Beyond data, the MDH’s second collaboration focus area was identifying resource needs for primary care practices and then working with other departments to meet those needs for clinicians. The MDH provided free point-of-care tests for clinicians to rapidly diagnose and determine therapeutic eligibility, as well as free personal protective equipment for staff. The MDH onboarded over 515 adult and family medicine primary care practices through March 31, 2022, 285 of which are in MDPCP, to accessibly deliver COVID-19 vaccines to patients and community members directly at each practice. As of March 2022, primary care practices in Maryland administered over 400 000 COVID-19 vaccine doses in office. As part of this work, the MDH also crafted resources and technical assistance packets to provide clinical and logistical support for primary care vaccine outreach. While the majority of MDPCP practices participated in administering vaccines directly in office, the remainder chose to refer to alternative vaccination sites.

The third focus area for integrating primary care in the public health response was providing consistent communication between the MDH and practices, filling an information vacuum. At the onset of the pandemic, the MDH hosted almost daily COVID-19 webinars to provide pandemic data, recommendations from the Centers for Disease Control and Prevention, US Food and Drug Administration authorizations, and clinical guidance for primary care clinicians. In accordance with COVID-19 surges, the webinars fluctuated in frequency. As of June 1, 2022, the MDH hosted over 120 webinars with more than 20 000 attendees.^15,16^ These webinars provided a consistent and trusted line of communication on urgent changes from state public health to primary care practices. In addition to webinars, the MDH also disseminated a weekly email that included topline messages, announcements, and updates on vaccines, testing, and therapeutics, and resources for practices and partners.^16^ Another communication focus area included information related to monoclonal antibodies. Monoclonal antibody therapy for the treatment of symptomatic high-risk COVID-19 was offered by over 50 Maryland sites, and all Maryland practices were informed of the location and referral mechanisms for these therapies.^17^

A final support was the allocation of population-based payments to MDPCP practices. Participation in the MDPCP transitions a practice’s Medicare fee-for-service payments to a value-based payment scheme, including non–visit-based population payments. When office visits declined dramatically in early 2020,^18^ non–fee-for-service financial resources from the MDPCP program enabled practices to retain staff and continuity of care. While the first 3 primary care integration response initiatives were open to both MDPCP and non-MDPCP practices, the availability of more flexible funding was only provided to practices in the MDPCP.

The MDPCP infrastructure enabled the MDH to integrate primary care into Maryland’s pandemic response. The MDH supported MDPCP practices in the COVID-19 response through the development of CRISP’s vaccine tracking system^13^ and other data reports, the identification and delivery of essential resources, a steady communication system, and non–volume-based financial resources. It is important to note that the first 3 COVID-19 integration initiatives were also open to non-MDPCP primary care practices, although participation consisted more heavily of MDPCP practices due to the existing relationship between the MDH and those participating practices.

## Methods

This study was deemed exempt from review and informed consent by the MDH Institutional Review Board because it constituted secondary research and used data at the aggregate level where individuals cannot be identified. The study followed the Strengthening the Reporting of Observational Studies in Epidemiology (STROBE) reporting guideline. All tabular data follow the CMS cell suppression policy.^19^

### Study Population

The study population was a subset of all Maryland Medicare fee-for-service beneficiaries, narrowed down to only those beneficiaries eligible for attribution to MDPCP continuously during the study period.^20^ Eligibility criteria included having continuous Part A and B coverage, not being incarcerated, not residing in a long-term care facility, not having end-stage kidney disease, and receiving the plurality of primary care or select primary care services from an eligible clinician specialty type.^20^ Data on beneficiary race and ethnicity were dervied from Medicare enrollment data, and these data were used to ensure matching of comparable demographics of beneficiary populations and because COVID-19 has impacted different racial and ethnic groups differentially.

This larger study population was divided into 2 distinct comparison groups: (1) beneficiaries whose attributed practice opted to participate in the MDPCP and (2) beneficiaries whose attributed practice opted not to participate. These groups are hereafter referred to as the MDPCP group and the nonparticipating group, respectively. By ensuring that both study populations have established relationships with primary care practices, the study aimed to isolate the association of MDPCP participation, rather than the association of a primary care relationship.

### Data Sources

The primary data source used for this analysis was Medicare Claim and Claim Line Feed data. This data set was combined with COVID-19 vaccination data from ImmuNet.^14^ Claims from January 1, 2020, through October 31, 2021, with a run-out period through January 31, 2022, and vaccination data from January 1, 2020, through March 31, 2022, were included in the analysis. Additionally, a data set from Socially Determined Inc containing COVID-19 Vulnerability Index (CVI) scores for each beneficiary was included to represent the beneficiary-level COVID-19 risk. Scores on the CVI ranged from 1 to 5, with 1 indicating lowest risk of severe COVID-19 and 5 indicating highest risk.

### Matching

To effectively examine the association of participation in the MDPCP on COVID-19 outcomes and compare outcomes in similar populations, the study and comparison groups were matched on several characteristics. A forced-match design matched the nonparticipating group to the MDPCP group on age category, sex, race and ethnicity, Medicare-Medicaid dual eligibility status, CVI score, and Maryland county of residence.

Matching was completed by assigning each beneficiary to 1 of the 5277 permutations that combine sex, age, race and ethnicity, county of residence, CVI, and dual eligibility values. For Maryland county of residence, 15 of Maryland’s largest jurisdictions based on MDPCP beneficiary population were matched directly, and the remaining counties were combined into an “other” category. Permutations with fewer than 18 beneficiaries were then removed from the data. From the remaining subcategories, beneficiaries were randomly selected from the nonparticipating population until the differences in distributions within each matched category were not significant using the χ^2^ statistical test. Prematch populations are shown in eTable 1 in Supplement 1.

To confirm the matching algorithm was effective in producing comparable populations, the same matching algorithm was utilized for a separate analysis comparing both in-hospital and all-cause mortality between the MDPCP group and nonparticipating group in 2019. This analysis found that 2019 mortality rates were not statistically different in these 2 groups, confirming that the matching algorithm yields comparison groups with the same pre-COVID mortality risk and is free from unmeasured confounding bias (eTable 2 in Supplement 1).

### Statistical Analysis

We used χ^2^ tests or 2-tailed *t* tests for normally distributed data and Mann-Whitney *U* tests for non–normally distributed data to compare demographic and clinical characteristics between the MDPCP group and nonparticipating group to determine any baseline characteristic differences. The following characteristic variables were compared: age category, sex, race and ethnicity, Medicare-Medicaid dual eligibility status, CVI score, Maryland county of residence, median Hierarchical Condition Category (HCC) score, and median Area Deprivation Index (ADI) score.^21^

We compared primary outcomes between the MDPCP group and nonparticipating group to determine any differences in COVID-19–related clinical care actions taken. This included variables related to vaccine status, vaccination status (not vaccinated, partially vaccinated, fully vaccinated with booster, fully vaccinated without booster), and booster vaccine status (yes or no). We also examined the vaccination rate over time between the 2 groups, comparing monthly vaccination rates in each group from December 1, 2020, to March 31, 2022. Additional primary outcome variables beyond vaccination status included treatment with monoclonal antibody infusions and percentage of COVID-19 beneficiaries with telehealth claims. Monoclonal antibody infusions were only counted for COVID-19–positive beneficiaries.

We also measured differences in clinical COVID-19 outcomes across the 2 groups using χ^2^ tests and *z* tests as secondary outcomes. These secondary outcome measures included percentage of beneficiaries with a COVID-19 diagnosis, percentage of beneficiaries with a COVID-19 inpatient claim, percentage of beneficiaries with a COVID-19 emergency department claim, percentage of beneficiaries who died of COVID-19, and the median COVID-19 inpatient admission length of stay.

Exploratory statistical analyses were replicated across each race and ethnicity subpopulation within the study groups. Analyses were conducted with SAS software, version 9.2 (SAS Institute Inc). Statistical significance was set at *P* < .05 for all statistical tests, and all tests were 2 tailed.

## Results

### Demographic and Clinical Characteristics

After matching, a total of 208 146 beneficiaries in the MDPCP group and 37 203 beneficiaries in the nonparticipating group were included, both comprising 60.10% women and 39.90% men with a median age of 76 (IQR, 71-82) years. Groups were compared across demographic and risk measures to determine any baseline differences. There were no significant differences between the MDPCP group and the nonparticipating group in terms of age category, sex, race and ethnicity, Medicare-Medicaid dual eligibility, CVI score, or Maryland county of residence (Table 1). Among participants in the MDPCP group and nonparticipating group, 1.22% and 1.23%, respectively, were Asian; 17.95% and 17.96%, respectively, were Black; 0.40% and 0.40%, respectively, were Hispanic; 78.40% and 78.38%, respectively, were White; and 0.80% and 0.81%, respectively, were of other race or ethnicities (specific categories unavailable).

**Table 1.** Characteristics of the Matched MDPCP Group and Nonparticipating Group

Characteristic	Participant group^a^		*P* value
	MDPCP (n = 208 146)	Nonparticipating (n = 37 203)	
Age category, y			
≤64	13 407 (6.44)	2389 (6.42)	>.99
65-69	22 967 (11.03)	4111 (11.05)	
70-74	56 307 (27.05)	10 060 (27.04)	
75-79	47 722 (22.93)	8534 (22.94)	
80-84	32 504 (15.62)	5808 (15.61)	
≥85	35 239 (16.93)	6301 (16.94)	
Sex			
Female	125 096 (60.10)	22 359 (60.10)	>.99
Male	83 050 (39.90)	14 844 (39.90)	
Race and ethnicity			
Asian	2544 (1.22)	458 (1.23)	>.99
Black	37 368 (17.95)	6680 (17.96)	
Hispanic	840 (0.40)	150 (0.40)	
White	163 194 (78.40)	29 161 (78.38)	
Other^b^	1664 (0.80)	300 (0.81)	
Unknown	2536 (1.22)	454 (1.22)	
Dual Medicare-Medicaid eligibility flag			
No	188 379 (90.50)	33 664 (90.49)	.92
Yes	19 767 (9.50)	3539 (9.51)	
CVI score^c^			
1 or 2	31 743 (15.25)	5672 (15.25)	>.99
3	64 513 (30.99)	11 519 (30.96)	
4	66 760 (32.07)	11 936 (32.08)	
5	45 130 (21.68)	8076 (21.71)	
County			
Anne Arundel	21 058 (10.12)	3763 (10.11)	>.99
Baltimore	35 055 (16.84)	6263 (16.83)	
Baltimore City	17 468 (8.39)	3121 (8.39)	
Calvert	4448 (2.14)	794 (2.13)	
Carroll	9398 (4.52)	1680 (4.52)	
Charles	4129 (1.98)	737 (1.98)	
Frederick	10 947 (5.26)	1957 (5.26)	
Harford	11 417 (5.49)	2039 (5.48)	
Howard	11 681 (5.61)	2087 (5.61)	
Montgomery	26 511 (12.74)	4738 (12.74)	
Other	20 777 (9.98)	3720 (10.00)	
Prince George’s	15 054 (7.23)	2693 (7.24)	
Saint Mary’s	4707 (2.26)	841 (2.26)	
Washington	7725 (3.71)	1379 (3.71)	
Wicomico	4114 (1.98)	737 (1.98)	
Worcester	3657 (1.76)	654 (1.76)	
HCC score, median (IQR)^d^	0.943 (0.568-1.598)	0.948 (0.573-1.632)	.007
ADI score, median (IQR)^e^	27 (16-42)	26 (15-41)	<.001

Abbreviations: ADI, Area Deprivation Index; CVI, COVID-19 Vulnerability Index; HCC, Hierarchical Condition Category; MDPCP, Maryland Primary Care Program.

^a^ Unless otherwise indicated, data are expressed as No. (%) of participants. Percentages have been rounded and therefore may not sum to 100.

^b^ Specific race and ethnicity categories are unavailable.

^c^ A score of 1 indicates lowest risk of severe COVID-19; a score of 5 indicates highest risk.

^d^ Scores range from 0 to no upper boundary, with higher scores indicating higher medical complexity.

^e^ Scores range from 0 to 100, with higher scores indicating higher neighborhood socioeconomic disadvantage.

The 2 groups did differ, however, in median (IQR) HCC score (0.943 [0.568-1.598] vs 0.948 [0.573-1.632]; *P* < .001) and median (IQR) ADI score (27 [16-42] vs 26 [15-41]; *P* < .001), indicating statistically significant differences in clinical risk and social risk (Table 1). However, the differences in HCC and ADI scores were small in magnitude, indicating that the populations in question are not meaningfully different in terms of clinical or social risk. In addition, these differences are directionally opposite, with the MDPCP group having a lower clinical risk score (HCC), but a higher social risk score (ADI).

### Primary COVID-19 Outcomes

The MDPCP group was shown to have associations of higher uptake of COVID-19 vaccination, monoclonal antibody infusions, and telehealth services used compared with the nonparticipating group. While 84.47% of the MDPCP group was fully vaccinated, 77.93% of the nonparticipating group was fully vaccinated (6.5–percentage point difference; *P* < .001). Higher levels of booster dose uptake were also seen in the MDPCP group compared with the nonparticipating group, with 65.80% in the MDPCP group and 58.51% in the nonparticipating group receiving a booster (12.4% higher rate; *P* < .001) (Table 2).

**Table 2.** Comparison of Uptake of COVID-19 Primary Outcomes Between the Matched MDPCP Group and Nonparticipating Group

Measure	Participant group^a^		*P* value
	MDPCP (n = 208 146)	Nonparticipating (n = 37 203)	
Vaccine status			
Not vaccinated	27 328 (13.13)	6926 (18.62)	<.001
Fully vaccinated			
With booster	136 958 (65.80)	21 768 (58.51)	
Without booster	38 855 (18.67)	7225 (19.42)	
Partially vaccinated	5005 (2.40)	1284 (3.45)	
Booster vaccine status			
No	71 188 (34.20)	15 435 (41.49)	<.001
Yes	136 958 (65.80)	21 768 (58.51)	
COVID-19–positive beneficiaries			
With monoclonal antibody infusion^b^	1152 (8.45)	161 (6.11)	<.001
With telehealth service claims^b^	8579 (62.95)	1438 (54.53)	<.001

Abbreviation: MDPCP, Maryland Primary Care Program.

^a^ Unless otherwise indicated, data are expressed as No. (%) of participants. Percentages have been rounded and therefore may not sum to 100.

^b^ Includes 13 628 in the MDPCP group and 2673 in the nonparticipating group.

In addition to studying the overall vaccination rate of the 2 populations, we analyzed differences in vaccination rates in each month from December 1, 2020, to March 31, 2022, to understand the change in vaccination rate over time in each population as vaccine supply increased. As shown in the Figure, the MDPCP group showed higher rates of vaccination than the nonparticipating group in every month of the study period.

**Figure.**
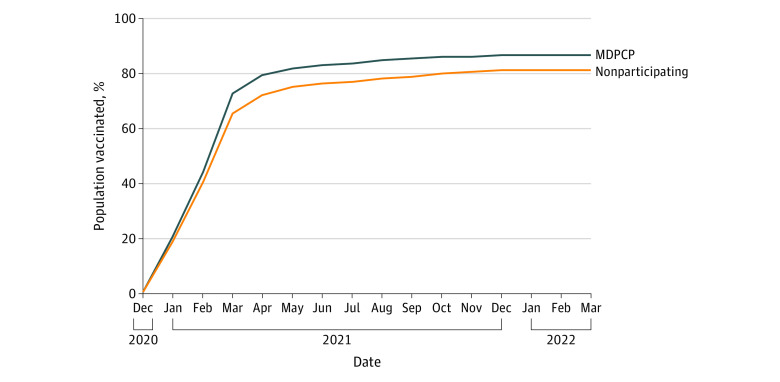
Vaccination Rates Over Time for Maryland Primary Care Program (MDPCP) and Nonparticipating Practice Groups

Comparing monoclonal antibody infusion rates, 8.45% of COVID-19–positive beneficiaries in the MDPCP group received treatment, compared with 6.11% of COVID-19–positive beneficiaries in the nonparticipating group (*P* < .001) (Table 2). Comparing telehealth service utilization, 62.95% of COVID-19–positive beneficiaries in the MDPCP group received care via telehealth, compared with 54.53% of the nonparticipating group (*P* < .001) (Table 2).

### Secondary COVID-19 Outcomes

Largely as a result of the differences in vaccination rates, beneficiaries in the MDPCP group had 7.6% lower rates of COVID-19 cases (6.55% vs 7.09%; *P* < .001), 12% lower rates of COVID-19 inpatient admissions (1.81% vs 2.06%; *P* = .001), and 27% lower rates of death due to COVID-19 (0.56% vs 0.77%; *P* < .001) compared with the nonparticipating group (Table 3). There was no difference between the MDPCP group and the nonparticipating group in rates of COVID-19 emergency department visits or median COVID-19 inpatient admission length of stay (Table 3). In subanalyses comparing the MDPCP group and nonparticipating group within racial and ethnic subpopulations, the same trends persisted, although the differences were not always statistically significant (eTables 3-20 in Supplement 1).

**Table 3.** COVID-19 Secondary Outcomes in the Matched MDPCP Group and Nonparticipating Group

Measure	Participant group^a^		*P* value
	MDPCP (n = 208 146)	Nonparticipating (n = 37 203)	
COVID-19–positive beneficiaries	13 628 (6.55)	2637 (7.09)	<.001
Beneficiaries with COVID-19			
Inpatient claims	3775 (1.81)	766 (2.06)	.001
Emergency department claims	2134 (1.03)	406 (1.09)	.25
COVID-19 death count	1168 (0.56)	285 (0.77)	<.001
COVID-19 inpatient admission length of stay, median (IQR), d	7 (5-11)	7 (5-12)	.08

Abbreviation: MDPCP, Maryland Primary Care Program.

^a^ Unless otherwise indicated, data are expressed as No. (%) of participants. Percentages have been rounded and may not total 100.

## Discussion

### Study Results

This cohort study analyzed more than 2 years of data, finding that Maryland Medicare beneficiaries in the MDPCP group displayed lower rates of COVID-19 cases, COVID-19 inpatient admissions, and deaths due to COVID-19 compared with the nonparticipating group. These data support the findings of the previous study.^10^ Data for Maryland Medicare beneficiaries in the MDPCP group include all participating MDPCP practices. This study showed COVID-19 inpatient admissions were 12% lower in the MDPCP group, and the COVID-19-attributed death rate of the MDPCP group was 27% lower than in the nonparticipating group. Consistent technical assistance, data tools, financial flexibility, and guidance from the MDH to primary care practices enabled better vaccination rates, therapeutics referrals, and telehealth provision, all enabling better health outcomes.

Despite matching of populations on factors including geography that may impact vaccination rates, vaccination rates were significantly higher in the MDPCP group compared with the nonparticipating group and included a 6.5–percentage point difference between groups. Boosters exhibited a similar difference, with a 12.4% higher booster uptake rate in the MDPCP group compared with the nonparticipating group. Given the demographic matching between the 2 comparison groups, the stark difference in vaccination rates in the 2 populations is likely a primary reason for better COVID-19 outcomes in the MDPCP group, as COVID-19 vaccines are the best protector against severe COVID-19 outcomes.^22,23,24^ The same directionality is true for monoclonal antibody infusions and telehealth services used, indicating that increased referrals for therapeutics and provision of care via telehealth in MDPCP practices contributed to better COVID-19 clinical outcomes during the study period.^25,26^

### Implications

Through the provision of data tools and dashboards, the rapid distribution of resources such as vaccines and point-of-care tests, the continuous communication of public health information from the state public health department to primary care practices, and the non–visit-based financial payments, Maryland’s collaborative model between public health and MDPCP advanced primary care practices showed improved outcomes for patients with COVID-19. This model of integration can be replicated in other states and localities to build preparedness for future public health emergencies.

A key success factor for Maryland’s collaborative model was the establishment of a relationship between primary care and public health before the COVID-19 pandemic began. This was evident in the vaccine rollout, where MDPCP practices were first to engage in receiving vaccine allocations, as they could be mobilized through existing communication channels.

Maryland’s success additionally showed the importance of public health providing data tools to the frontline health care sector, especially in a crisis. Public health maintains state immunization records and was able to compile and display these data for primary care practices to facilitate targeted and efficient patient immunizations. Integrating primary care and public health has larger implications beyond COVID-19. As known and emerging infectious diseases are expected to increase in frequency,^27^ this model can be replicated to efficiently engage primary care into public health crisis responses. For common infectious disease threats such as influenza, similar public health data tools can be mobilized to improve vaccination rates in vulnerable populations. Even beyond infectious diseases, this model can be applied in other public health focus areas such as chronic diseases and behavioral health. For example, public health can communicate with primary care on state-based diabetes management resources for referral and provide data tools for understanding broader regional trends in diabetes prevalence and control. The integration of primary care within the state public health system in Maryland can be a model for other states to more effectively achieve key public health priorities.

### Limitations

This study has some limitations. The MDPCP is a voluntary program for primary care practices, and it is not known whether there are any preexisting differences between practices that choose to participate and those that do not. Any selection bias for participating in the MDPCP could factor into differences in COVID-19 primary and secondary outcomes. Due to a lack of data on practice characteristics for nonparticipating practices, it is not known whether MDPCP practices have underlying structural differences (eg, more or less health system affiliation, differences in practice size) compared with nonparticipating practices. Matching on many descriptive variables, including geography and beneficiary demographic makeup, eliminates obvious differences between MDPCP and nonparticipating populations and likely minimizes the effects of any potential practice selection bias.

The time horizon of analysis of vaccine data in this study runs through March 2022, whereas claims data run through October 2021. The reason for this discrepancy is that claims data have a months-long run-out period before claims information is final and complete. Immunization data do not have a run-out period and can therefore be more timely. Because of lack of claims data between November 2021 and March 2022, the link between differences in vaccination rates between MDPCP and nonparticipating beneficiaries and COVID-19 outcomes in this time period cannot be made. However, the link between vaccination and COVID-19 outcomes data is clear between December 2020 and October 2021 and is expected to persist over time.

Additionally, the use of claims data to determine COVID-19 cases excludes any beneficiaries who did not seek health care services for their COVID-19 illness and thus did not generate a claim. As such, the COVID-19 case rate is likely an undercount. In parallel, only COVID-19 deaths that generate a Medicare claim are included. This error is systemic and is likely to have similar effects on the MDPCP and nonparticipating groups.

## Conclusions

The findings of this cohort study suggest that utilization rates of vaccines, monoclonal antibodies, and telehealth care services were higher among MDPCP beneficiaries compared with a matched cohort. In parallel, participation in the MDPCP was also associated with less adverse COVID-19 outcomes, including rates of cases, hospitalizations, and deaths. The coordinated partnership between state public health and primary care practices enabled the provision of support, technical assistance, data tools, and swift communication with MDPCP practices. This partnership was associated with better uptake of COVID-19 vaccines, monoclonal antibodies, and telehealth services and ultimately better COVID-19 outcomes.

## Notes

Supplement 1. **eTable 1.** MDPCP and Nonparticipating Populations Before Matching **eTable 2.** Comparison of 2019 In-Hospital and All-Cause Mortality Using the Same Matching Algorithm as the Main Analysis **eTable 3.** Demographic and Risk Characteristic Comparison Between the Matched MDPCP Group and Nonparticipating Group, for Asian Beneficiaries Only **eTable 4.** Comparison of Uptake of COVID-19 Primary Outcomes Between the Matched MDPCP Group and Nonparticipating Group, for Asian Beneficiaries Only **eTable 5.** COVID-19 Secondary Outcomes in the Matched MDPCP Group and Nonparticipating Group, for Asian Beneficiaries Only **eTable 6.** Demographic and Risk Characteristic Comparison Between the Matched MDPCP Group and Nonparticipating Group, for Black Beneficiaries Only **eTable 7.** Comparison of Uptake of COVID-19 Primary Outcomes Between the Matched MDPCP Group and Nonparticipating Group, for Black Beneficiaries Only **eTable 8**. COVID-19 Secondary Outcomes in the Matched MDPCP Group and Nonparticipating Group, for Black Beneficiaries Only **eTable 9.** Demographic and Risk Characteristic Comparison Between the Matched MDPCP Group and Nonparticipating Group, for Hispanic Beneficiaries Only **eTable 10.** Comparison of Uptake of COVID-19 Primary Outcomes Between the Matched MDPCP Group and Nonparticipating Group, for Hispanic Beneficiaries Only **eTable 11.** COVID-19 Secondary Outcomes in the Matched MDPCP Group and Nonparticipating Group, for Hispanic Beneficiaries Only **eTable 12.** Demographic and Risk Characteristic Comparison Between the Matched MDPCP Group and Nonparticipating Group, for Other Race and Ethnicity Beneficiaries Only **eTable 13.** Demographic and Risk Characteristic Comparison Between the Matched MDPCP Group and Nonparticipating Group, for Other Race and Ethnicity Beneficiaries Only **eTable 14.** COVID-19 Secondary Outcomes in the Matched MDPCP Group and Nonparticipating Group, for Other Race and Ethnicity Beneficiaries Only **eTable 15.** COVID-19 Secondary Outcomes in the Matched MDPCP Group and Nonparticipating Group, for Other Race and Ethnicity Beneficiaries Only **eTable 16.** Comparison of Uptake of COVID-19 Primary Outcomes Between the Matched MDPCP Group and Nonparticipating Group, for Unknown Race and Ethnicity Beneficiaries Only **eTable 17.** COVID-19 Secondary Outcomes in the Matched MDPCP Group and Nonparticipating Group, for Unknown Race and Ethnicity Beneficiaries Only **eTable 18.** Demographic and Risk Characteristic Comparison Between the Matched MDPCP Group and Nonparticipating Group, for White Beneficiaries Only **eTable 19.** Comparison of Uptake of COVID-19 Primary Outcomes Between the Matched MDPCP Group and Nonparticipating Group, for White Race Beneficiaries Only **eTable 20.** COVID-19 Secondary Outcomes in the Matched MDPCP Group and Nonparticipating Group, for White Race Beneficiaries Only

Supplement 2.
Data Sharing Statement
