# Supplementary material for: Association of Participation in the Maryland Primary Care Program With COVID-19 Outcomes Among Medicare Beneficiaries
Source: JAMA Netw Open. 2023 Jan 6;6(1):e2249791. doi: 10.1001/jamanetworkopen.2022.49791 (PMC9856987; doi:10.1001/jamanetworkopen.2022.49791)
Supplement: Supplement 1. — eTable 1. MDPCP and Nonparticipating Populations Before Matching eTable 2. Comparison of 2019 In-Hospital and All-Cause Mortality Using the Same Matching Algorithm as the Main Analysis eTable 3. Demographic and Risk Characteristic Comparison Between the Matched MDPCP Group and Nonparticipating Group, for Asian Beneficiaries Only eTable 4. Comparison of Uptake of COVID-19 Primary Outcomes Between the Matched MDPCP Group and Nonparticipating Group, for Asian Beneficiaries Only eTable 5. COVID-19 Secondary Outcomes in the Matched MDPCP Group and Nonparticipating Group, for Asian Beneficiaries Only eTable 6. Demographic and Risk Characteristic Comparison Between the Matched MDPCP Group and Nonparticipating Group, for Black Beneficiaries Only eTable 7. Comparison of Uptake of COVID-19 Primary Outcomes Between the Matched MDPCP Group and Nonparticipating Group, for Black Beneficiaries Only eTable 8. COVID-19 Secondary Outcomes in the Matched MDPCP Group and Nonparticipating Group, for Black Beneficiaries Only eTable 9. Demographic and Risk Characteristic Comparison Between the Matched MDPCP Group and Nonparticipating Group, for Hispanic Beneficiaries Only eTable 10. Comparison of Uptake of COVID-19 Primary Outcomes Between the Matched MDPCP Group and Nonparticipating Group, for Hispanic Beneficiaries Only eTable 11. COVID-19 Secondary Outcomes in the Matched MDPCP Group and Nonparticipating Group, for Hispanic Beneficiaries Only eTable 12. Demographic and Risk Characteristic Comparison Between the Matched MDPCP Group and Nonparticipating Group, for Other Race and Ethnicity Beneficiaries Only eTable 13. Demographic and Risk Characteristic Comparison Between the Matched MDPCP Group and Nonparticipating Group, for Other Race and Ethnicity Beneficiaries Only eTable 14. COVID-19 Secondary Outcomes in the Matched MDPCP Group and Nonparticipating Group, for Other Race and Ethnicity Beneficiaries Only eTable 15. COVID-19 Secondary Outcomes in the Matched MDPCP Group and Nonpartic [file jamanetwopen-e2249791-s001.pdf]

Supplementary Online Content

Gruber E, Perman C, Grisham R, Adashi EY, Haft H. Association of participation in the Maryland Primary Care Program with COVID-19 outcomes among Medicare beneficiaries. *JAMA Netw Open*. 2023;6(1):e2249791. doi:10.1001/jamanetworkopen.2022.49791

eTable 1. MDPCP and Nonparticipating Populations Before Matching
eTable 2. Comparison of 2019 In-Hospital and All-Cause Mortality Using the Same Matching Algorithm as the Main Analysis
eTable 3. Demographic and Risk Characteristic Comparison Between the Matched MDPCP Group and Nonparticipating Group, for Asian Beneficiaries Only
eTable 4. Comparison of Uptake of COVID-19 Primary Outcomes Between the Matched MDPCP Group and Nonparticipating Group, for Asian Beneficiaries Only
eTable 5. COVID-19 Secondary Outcomes in the Matched MDPCP Group and Nonparticipating Group, for Asian Beneficiaries Only
eTable 6. Demographic and Risk Characteristic Comparison Between the Matched MDPCP Group and Nonparticipating Group, for Black Beneficiaries Only
eTable 7. Comparison of Uptake of COVID-19 Primary Outcomes Between the Matched MDPCP Group and Nonparticipating Group, for Black Beneficiaries Only
eTable 8. COVID-19 Secondary Outcomes in the Matched MDPCP Group and Nonparticipating Group, for Black Beneficiaries Only
eTable 9. Demographic and Risk Characteristic Comparison Between the Matched MDPCP Group and Nonparticipating Group, for Hispanic Beneficiaries Only
eTable 10. Comparison of Uptake of COVID-19 Primary Outcomes Between the Matched MDPCP Group and Nonparticipating Group, for Hispanic Beneficiaries Only
eTable 11. COVID-19 Secondary Outcomes in the Matched MDPCP Group and Nonparticipating Group, for Hispanic Beneficiaries Only
eTable 12. Demographic and Risk Characteristic Comparison Between the Matched MDPCP Group and Nonparticipating Group, for Other Race and Ethnicity Beneficiaries Only
eTable 13. Demographic and Risk Characteristic Comparison Between the Matched MDPCP Group and Nonparticipating Group, for Other Race and Ethnicity Beneficiaries Only
eTable 14. COVID-19 Secondary Outcomes in the Matched MDPCP Group and Nonparticipating Group, for Other Race and Ethnicity Beneficiaries Only
eTable 15. COVID-19 Secondary Outcomes in the Matched MDPCP Group and Nonparticipating Group, for Other Race and Ethnicity Beneficiaries Only
eTable 16. Comparison of Uptake of COVID-19 Primary Outcomes Between the Matched MDPCP Group and Nonparticipating Group, for Unknown Race and Ethnicity Beneficiaries Only
eTable 17. COVID-19 Secondary Outcomes in the Matched MDPCP Group and Nonparticipating Group, for Unknown Race and Ethnicity Beneficiaries Only
eTable 18. Demographic and Risk Characteristic Comparison Between the Matched MDPCP Group and Nonparticipating Group, for White Beneficiaries Only
eTable 19. Comparison of Uptake of COVID-19 Primary Outcomes Between the Matched MDPCP Group and Nonparticipating Group, for White Race Beneficiaries Only
eTable 20. COVID-19 Secondary Outcomes in the Matched MDPCP Group and Nonparticipating Group, for White Race Beneficiaries Only

This supplementary material has been provided by the authors to give readers additional information about their work.

© 2023 Gruber E et al. *JAMA Network Open*.

eTable 1. MDPCP and Nonparticipating Populations Before Matching

Measures	MDPCP Group (n = 230,160) n (%)	Non-Participating Group (n = 168,547) n (%)	p-value
**Age Category**			<.001
64 and Younger	16,151 (7.02)	18,295 (10.85)	
65 to 69	26,732 (11.61)	19,416 (11.52)	
70 to 74	61,287 (26.63)	41,629 (24.7)	
75 to 79	52,141 (22.65)	35,304 (20.95)	
80 to 84	35,898 (15.6)	25,035 (14.85)	
85 and Older	37,951 (16.49)	28,868 (17.13)	
**Gender**			.38
Female	136,794 (59.43)	100,407 (59.57)	
Male	93,366 (40.57)	68,140 (40.43)	
**Race**			<.001
Asian	4,487 (1.95)	6,705 (3.98)	
Black	45,385 (19.72)	38,854 (23.05)	
Hispanic	1,905 (0.83)	2,164 (1.28)	
Other	3,751 (1.63)	3,370 (2)	
Unknown	5,478 (2.38)	4,324 (2.57)	
White	169,154 (73.49)	113,130 (67.12)	
**Dual Medicare-Medicaid Eligibility Flag**			<.001
No	201,401 (87.5)	136,236 (80.83)	
Yes	28,759 (12.5)	32,311 (19.17)	
**COVID-19 Vulnerability Index Score**			<.001
1 or 2	36,824 (16)	39,758 (23.59)	
3	70,488 (30.63)	45,872 (27.22)	
4	73,173 (31.79)	48,305 (28.66)	
5	49,675 (21.58)	34,612 (20.54)	
**County**			<.001
Allegany	22,492 (9.77)	14,129 (8.38)	
Anne Arundel	36,878 (16.02)	19,433 (11.53)	
Baltimore	18,608 (8.08)	10,773 (6.39)	
Baltimore City	5,685 (2.47)	1,948 (1.16)	
Calvert	10,584 (4.6)	4,302 (2.55)	
Carroll	5,038 (2.19)	4,891 (2.9)	
Charles	12,620 (5.48)	5,343 (3.17)	
Frederick	13,798 (5.99)	4,358 (2.59)	

© 2023 Gruber E et al. *JAMA Network Open*.

Harford	13,743 (5.97)	5,953 (3.53)	
Howard	27,880 (12.11)	28,787 (17.08)	
Montgomery	21,361 (9.28)	35,087 (20.82)	
Other	16,037 (6.97)	21,467 (12.74)	
Prince Georges	6,760 (2.94)	2,080 (1.23)	
Washington	8,527 (3.7)	5,189 (3.08)	
Wicomico	5,440 (2.36)	2,978 (1.77)	
Worcester	4,709 (2.05)	1,829 (1.09)	
**Average HCC Score**			<.001
	1.3751	1.4248	
**Average ADI Score**			.02
	31.41	31.58	

© 2023 Gruber E et al. *JAMA Network Open*.

eTable 2. Comparison of 2019 In-Hospital and All-Cause Mortality Using the Same Matching Algorithm as the Main Analysis

	**MDPCP Group (n = 195,013)**	**Non-Participating Group (n = 74,980)**	
**Measures**	**n (%)**	**n (%)**	**p-value**
**Age Category**			>.99
64 and Younger	15,349 (7.87)	5,896 (7.86)	
65 to 69	26,922 (13.81)	10,353 (13.81)	
70 to 74	52,417 (26.88)	20,157 (26.88)	
75 to 79	41,419 (21.24)	15,918 (21.23)	
80 to 84	27,747 (14.23)	10,667 (14.23)	
85 and Older	31,159 (15.98)	11,989 (15.99)	
**Gender**			>.99
Female	117,325 (60.16)	45,109 (60.16)	
Male	77,688 (39.84)	29,871 (39.84)	
**Race**			>.99
Asian	2,791 (1.43)	1,071 (1.43)	
Black	39,263 (20.13)	15,084 (20.12)	
Hispanic	1,133 (0.58)	438 (0.58)	
Other	1,950 (1)	747 (1)	
Unknown	2,877 (1.48)	1,108 (1.48)	
White	146,999 (75.38)	56,532 (75.4)	
**Dual Medicare-Medicaid Eligibility Flag**			.95
No	173,533 (88.99)	66,727 (88.99)	
Yes	21,480 (11.01)	8,253 (11.01)	
**COVID-19 Vulnerability Index Score**			>.99
1 or 2	35,428 (18.17)	13,624 (18.17)	
3	55,482 (28.45)	21,326 (28.44)	
4	60,515 (31.03)	23,275 (31.04)	
5	43,588 (22.35)	16,755 (22.35)	
**County**			>.99
Allegany	3,306 (1.7)	1,266 (1.69)	
Anne Arundel	17,355 (8.9)	6,677 (8.91)	
Baltimore	29,131 (14.94)	11,201 (14.94)	
Baltimore City	19,001 (9.74)	7,302 (9.74)	
Calvert	4,597 (2.36)	1,770 (2.36)	
Carroll	8,864 (4.55)	3,408 (4.55)	

© 2023 Gruber E et al. *JAMA Network Open*.

Charles	2,975 (1.53)	1,147 (1.53)	
Frederick	9,960 (5.11)	3,834 (5.11)	
Harford	9,725 (4.99)	3,740 (4.99)	
Howard	9,018 (4.62)	3,469 (4.63)	
Montgomery	30,187 (15.48)	11,600 (15.47)	
Other	18,516 (9.49)	7,118 (9.49)	
Prince Georges	16,241 (8.33)	6,247 (8.33)	
Washington	7,176 (3.68)	2,755 (3.67)	
Wicomico	5,007 (2.57)	1,923 (2.56)	
Worcester	3,954 (2.03)	1,523 (2.03)	
**Hospital Death Count**			.21
	1,890 (0.97)	687 (0.92)	
**All-Cause Death Count**			.90
	6,832 (3.5)	2,634 (3.51)	

© 2023 Gruber E et al. *JAMA Network Open*.

eTable 3. Demographic and Risk Characteristic Comparison Between the Matched MDPCP Group and Nonparticipating Group, for Asian Beneficiaries Only

	**MDPCP Group (n = 2,544)**	**Non-Participating Group (n = 458)**	
**Measures**	**n (%)**	**n (%)**	**p-value**
**Age Category**			>.99
64 and Younger	44 (1.73)	<11 (<2.4)	
65 to 69	241 (9.47)	44 (9.61)	
70 to 74	730 (28.69)	131 (28.6)	
75 to 79	583 (22.92)	105 (22.93)	
80 to 84	405 (15.92)	73 (15.94)	
85 and Older	541 (21.27)	>94 (>20.52)	
**Gender**			.89
Female	1,691 (66.47)	306 (66.81)	
Male	853 (33.53)	152 (33.19)	
**Dual Medicare-Medicaid Eligibility Flag**			.84
No	1,259 (49.49)	229 (50)	
Yes	1,285 (50.51)	229 (50)	
**COVID-19 Vulnerability Index Score**			>.99
1 or 2	168 (6.6)	31 (6.77)	
3	914 (35.93)	164 (35.81)	
4	919 (36.12)	164 (35.81)	
5	543 (21.34)	99 (21.62)	
**County**			>.990
Anne Arundel	33 (1.3)	<11 (<2.4)	
Baltimore	202 (7.94)	36 (7.86)	
Baltimore City	<11 (0.31)	<11 (<2.4)	
Howard	290 (11.4)	53 (11.57)	
Montgomery	1,850 (72.72)	332 (72.49)	
Other	<11 (0.08)	0	
Prince Georges	159 (6.25)	>15 (>3.28)	
**Average HCC Score**			.19
	1.3071	1.2231	
**Average ADI Score**			.57
	18.70	19.11	

© 2023 Gruber E et al. *JAMA Network Open*.

eTable 4. Comparison of Uptake of COVID-19 Primary Outcomes Between the Matched MDPCP Group and Nonparticipating Group, for Asian Beneficiaries Only

	**MDPCP Group (n = 2,544)**	**Non-Participating Group (n = 458)**	
**Measures**	**n (%)**	**n (%)**	**p-value**
**Vaccine Status**			.02
NOT vaccinated	301 (11.83)	68 (14.85)	
fully vaccinated with booster	1,762 (69.26)	284 (62.01)	
fully vaccinated without booster	408 (16.04)	87 (19)	
partially vaccinated	73 (2.87)	19 (4.15)	
**Booster Vaccine Status**			.002
No	782 (30.74)	174 (37.99)	
Yes	1,762 (69.26)	284 (62.01)	
**COVID-19 positive beneficiaries with monoclonal antibody infusion**	<11 (<8.46)	0 (0)	.44
**COVID-19 positive beneficiaries with telehealth service claims**	247 (63.85)	47 (66.67)	.39

© 2023 Gruber E et al. *JAMA Network Open*.

eTable 5. COVID-19 Secondary Outcomes in the Matched MDPCP Group and Nonparticipating Group, for Asian Beneficiaries Only

	MDPCP Group (n = 2,544)	Non-Participating Group (n = 458)	
**Measures**	**n (%)**	**n (%)**	**p-value**
COVID-19 Positive Beneficiaries	130 (5.11)	15 (3.28)	.09
Beneficiaries with COVID-19 inpatient claims	43 (1.69)	<11 (1.53)	.80
Beneficiaries with COVID-19 emergency department claims	25 (0.98)	<11 (0.87)	.83
COVID-19 Death Count	15 (0.59)	<11 (0.44)	.69
Average COVID-19 inpatient admission length of stay	9.58	7.18	.33

© 2023 Gruber E et al. *JAMA Network Open*.

eTable 6. Demographic and Risk Characteristic Comparison Between the Matched MDPCP Group and Nonparticipating Group, for Black Beneficiaries Only

	MDPCP Group (n = 37,368)	Non-Participating Group (n = 6,680)	
**Measures**	**n (%)**	**n (%)**	**p-value**
**Age Category**			>.99
64 and Younger	4,827 (12.92)	860 (12.87)	
65 to 69	4,611 (12.34)	828 (12.4)	
70 to 74	10,557 (28.25)	1,885 (28.22)	
75 to 79	7,491 (20.05)	1,339 (20.04)	
80 to 84	4,976 (13.32)	889 (13.31)	
85 and Older	4,906 (13.13)	879 (13.16)	
**Gender**			.96
Female	25,498 (68.23)	4,560 (68.26)	
Male	11,870 (31.77)	2,120 (31.74)	
**Dual Medicare-Medicaid Eligibility Flag**			.92
No	29,779 (79.69)	5,320 (79.64)	
Yes	7,589 (20.31)	1,360 (20.36)	
**COVID-19 Vulnerability Index Score**			>.99
1 or 2	5,862 (15.69)	1,047 (15.67)	
3	10,275 (27.5)	1,833 (27.44)	
4	12,392 (33.16)	2,217 (33.19)	
5	8,839 (23.65)	1,583 (23.7)	
**County**			>.99
Anne Arundel	2,288 (6.12)	410 (6.14)	
Baltimore	7,214 (19.31)	1,290 (19.31)	
Baltimore City	10,036 (26.86)	1,794 (26.86)	
Calvert	105 (0.28)	18 (0.27)	
Charles	1,364 (3.65)	244 (3.65)	
Frederick	68 (0.18)	12 (0.18)	
Harford	186 (0.5)	33 (0.49)	
Howard	1,183 (3.17)	211 (3.16)	
Montgomery	3,151 (8.43)	563 (8.43)	
Other	2,057 (5.5)	367 (5.49)	
Prince Georges	9,394 (25.14)	1,680 (25.15)	
Saint Marys	61 (0.16)	11 (0.16)	

© 2023 Gruber E et al. *JAMA Network Open*.

Washington	54 (0.14)	<11 (<0.16)	
Wicomico	207 (0.55)	>36 (>0.54)	
**Average HCC Score**			.33
	1.4462	1.4651	
**Average ADI Score**			.44
	42.46	42.72	

© 2023 Gruber E et al. *JAMA Network Open*.

eTable 7. Comparison of Uptake of COVID-19 Primary Outcomes Between the Matched MDPCP Group and Nonparticipating Group, for Black Beneficiaries Only

	MDPCP Group (n = 37,368)	Non-Participating Group (n = 6,680)	
**Measures**	**n (%)**	**n (%)**	**p-value**
**Vaccine Status**			<.001
NOT vaccinated	5,296 (14.17)	1,328 (19.88)	
fully vaccinated with booster	23,764 (63.59)	3,729 (55.82)	
fully vaccinated without booster	7,180 (19.21)	1,336 (20)	
partially vaccinated	1,128 (3.02)	287 (4.3)	
**Booster Vaccine Status**			<.001
No	13,604 (36.41)	2,951 (44.18)	
Yes	23,764 (63.59)	3,729 (55.82)	
**COVID-19 positive beneficiaries with monoclonal antibody infusion**	153 (5.18)	12 (2.28)	.004
**COVID-19 positive beneficiaries with telehealth service claims**	4,885 (66.01)	857 (54.84)	<.001

© 2023 Gruber E et al. *JAMA Network Open*.

eTable 8. COVID-19 Secondary Outcomes in the Matched MDPCP Group and Nonparticipating Group, for Black Beneficiaries Only

	**MDPCP Group (n = 37,368)**	**Non-Participating Group (n = 6,680)**	
**Measures**	**n (%)**	**n (%)**	**p-value**
COVID-19 Positive Beneficiaries	2,954 (7.91)	527 (7.89)	.96
Beneficiaries with COVID-19 inpatient claims	1,022 (2.73)	184 (2.75)	.93
Beneficiaries with COVID-19 emergency department claims	588 (1.57)	92 (1.38)	.23
COVID-19 Death Count	290 (0.78)	60 (0.9)	.30
Average COVID-19 inpatient admission length of stay	9.84	10.22	.60

© 2023 Gruber E et al. *JAMA Network Open*.

eTable 9. Demographic and Risk Characteristic Comparison Between the Matched MDPCP Group and Nonparticipating Group, for Hispanic Beneficiaries Only

	MDPCP Group (n = 840)	Non-Participating Group (n = 150)	
**Measures**	**n (%)**	**n (%)**	**p-value**
**Age Category**			>.99
64 and Younger	74 (8.81)	13 (8.67)	
65 to 69	79 (9.4)	14 (9.33)	
70 to 74	248 (29.52)	44 (29.33)	
75 to 79	149 (17.74)	27 (18)	
80 to 84	137 (16.31)	24 (16)	
85 and Older	153 (18.21)	28 (18.67)	
**Gender**			.79
Female	664 (79.05)	120 (80)	
Male	176 (20.95)	30 (20)	
**Dual Medicare-Medicaid Eligibility Flag**			.86
No	230 (27.38)	40 (26.67)	
Yes	610 (72.62)	110 (73.33)	
**COVID-19 Vulnerability Index Score**			>.99
1 or 2	102 (12.14)	18 (12)	
3	329 (39.17)	59 (39.33)	
4	264 (31.43)	46 (30.67)	
5	145 (17.26)	27 (18)	
**County**			>.99
Montgomery	728 (86.67)	130 (86.67)	
Prince Georges	112 (13.33)	20 (13.33)	
**Average HCC Score**			.39
	1.2746	1.1941	
**Average ADI Score**			.43
	23.01	22.10	

© 2023 Gruber E et al. *JAMA Network Open*.

eTable 10. Comparison of Uptake of COVID-19 Primary Outcomes Between the Matched MDPCP Group and Nonparticipating Group, for Hispanic Beneficiaries Only

	MDPCP Group (n = 840)	Non-Participating Group (n = 150)	
**Measures**	**n (%)**	**n (%)**	**p-value**
**Vaccine Status**			.43
NOT vaccinated	146 (17.38)	>27 (>18.01)	
fully vaccinated with booster	471 (56.07)	74 (49.33)	
fully vaccinated without booster	198 (23.57)	38 (25.33)	
partially vaccinated	25 (2.98)	<11 (<7.33)	
**Booster Vaccine Status**			.13
No	369 (43.93)	76 (50.67)	
Yes	471 (56.07)	74 (49.33)	
**COVID-19 positive beneficiaries with monoclonal antibody infusion**	<11 (3.51)	0	.27
**COVID-19 positive beneficiaries with telehealth service claims**	266 (80.7)	111 (73.53)	.78

© 2023 Gruber E et al. *JAMA Network Open*.

eTable 11. COVID-19 Secondary Outcomes in the Matched MDPCP Group and Nonparticipating Group, for Hispanic Beneficiaries Only

	**MDPCP Group (n = 840)**	**Non-Participating Group (n = 150)**	
**Measures**	**n (%)**	**n (%)**	**p-value**
COVID-19 Positive Beneficiaries	114 (13.57)	34 (22.67)	.004
Beneficiaries with COVID-19 inpatient claims	35 (4.17)	12 (8)	.04
Beneficiaries with COVID-19 emergency department claims	23 (2.74)	<11 (<7.33)	.31
COVID-19 Death Count	13 (1.55)	<11 (<7.33)	.69
Average COVID-19 inpatient admission length of stay	11.42	14.62	.49

© 2023 Gruber E et al. *JAMA Network Open*.

eTable 12. Demographic and Risk Characteristic Comparison Between the Matched MDPCP Group and Nonparticipating Group, for Other Race and Ethnicity Beneficiaries Only

	MDPCP Group (n = 1,664)	Non-Participating Group (n = 300)	
**Measures**	**n (%)**	**n (%)**	**p-value**
**Age Category**			>.99
64 and Younger	0	0	
65 to 69	59 (3.55)	11 (3.67)	
70 to 74	488 (29.33)	86 (28.67)	
75 to 79	478 (28.73)	86 (28.67)	
80 to 84	401 (24.1)	73 (24.33)	
85 and Older	238 (14.3)	44 (14.67)	
**Gender**			.98
Female	1,011 (60.76)	182 (60.67)	
Male	653 (39.24)	118 (39.33)	
**Dual Medicare-Medicaid Eligibility Flag**			.93
No	1,590 (95.55)	287 (95.67)	
Yes	74 (4.45)	13 (4.33)	
**COVID-19 Vulnerability Index Score**			.99
1 or 2	0	0	
3	586 (35.22)	105 (35)	
4	778 (46.75)	140 (46.67)	
5	300 (18.03)	55 (18.33)	
**County**			
Anne Arundel	32 (1.92)	<11 (<3.67)	
Baltimore	140 (8.41)	26 (8.67)	
Howard	148 (8.89)	26 (8.67)	
Montgomery	1,162 (69.83)	209 (69.67)	
Prince Georges	182 (10.94)	>28 (>9.32)	
**Average HCC Score**			.17
	1.1269	1.0415	
**Average ADI Score**			.06
	17.26	15.72	

© 2023 Gruber E et al. *JAMA Network Open*.

eTable 13. Comparison of Uptake of COVID-19 Primary Outcomes Between the Matched MDPCP Group and Nonparticipating Group, for Other Race and Ethnicity Beneficiaries Only

	**MDPCP Group (n = 1,664)**	**Non-Participating Group (n = 300)**	
**Measures**	**n (%)**	**n (%)**	**p-value**
**Vaccine Status**			.03
NOT vaccinated	109 (6.55)	31 (10.33)	
fully vaccinated with booster	1,274 (76.56)	224 (74.67)	
fully vaccinated without booster	222 (13.34)	30 (10)	
partially vaccinated	59 (3.55)	15 (5)	
**Booster Vaccine Status**			.48
No	390 (23.44)	76 (25.33)	
Yes	1,274 (76.56)	224 (74.67)	
**COVID-19 positive beneficiaries with monoclonal antibody infusion**	<11 (<16.92)	0 (0)	.35
**COVID-19 positive beneficiaries with telehealth service claims**	119 (69.23)	52 (66.67)	.88

© 2023 Gruber E et al. *JAMA Network Open*.

eTable 14. COVID-19 Secondary Outcomes in the Matched MDPCP Group and Nonparticipating Group, for Other Race and Ethnicity Beneficiaries Only

	MDPCP Group (n = 1,664)	Non-Participating Group (n = 300)	
**Measures**	**n (%)**	**n (%)**	**p-value**
COVID-19 Positive Beneficiaries	65 (3.91)	18 (6)	.10
Beneficiaries with COVID-19 inpatient claims	20 (1.2)	<11 (2)	.27
Beneficiaries with COVID-19 emergency department claims	<11 (0.42)	0 (0)	.26
COVID-19 Death Count	<11 (0.18)	0 (0)	.46
Average COVID-19 inpatient admission length of stay	10.21	12.83	.44

© 2023 Gruber E et al. *JAMA Network Open*.

eTable 15. Demographic and Risk Characteristic Comparison Between the Matched MDPCP Group and Nonparticipating Group, for Unknown Race and Ethnicity Beneficiaries Only

	**MDPCP Group (n = 2,536)**	**Non-Participating Group (n = 454)**	
**Measures**	**n (%)**	**n (%)**	**p-value**
**Age Category**			>.99
64 and Younger	26 (1.03)	<11 (<2.42)	
65 to 69	320 (12.62)	58 (12.78)	
70 to 74	1,880 (74.13)	335 (73.79)	
75 to 79	204 (8.04)	37 (8.15)	
80 to 84	58 (2.29)	>2 (>0.44)	
85 and Older	48 (1.89)	<11 (<2.42)	
**Gender**			.88
Female	998 (39.35)	177 (38.99)	
Male	1,538 (60.65)	277 (61.01)	
**Dual Medicare-Medicaid Eligibility Flag**			.94
No	2,215 (87.34)	396 (87.22)	
Yes	321 (12.66)	58 (12.78)	
**COVID-19 Vulnerability Index Score**			.98
1 or 2	397 (15.65)	>70 (>15.42)	
3	1,568 (61.83)	278 (61.23)	
4	528 (20.82)	95 (20.93)	
5	43 (1.7)	<11 (<2.42)	
**County**			>.99
Anne Arundel	240 (9.46)	43 (9.47)	
Baltimore	469 (18.49)	83 (18.28)	
Baltimore City	114 (4.5)	20 (4.41)	
Frederick	71 (2.8)	13 (2.86)	
Howard	217 (8.56)	38 (8.37)	
Montgomery	996 (39.27)	179 (39.43)	
Other	319 (12.58)	58 (12.78)	
Prince Georges	110 (4.34)	20 (4.41)	
**Average HCC Score**			.53
	0.9164	0.8891	
**Average ADI Score**			.05

© 2023 Gruber E et al. *JAMA Network Open*.

	20.26	18.64	

© 2023 Gruber E et al. *JAMA Network Open*.

eTable 16. Comparison of Uptake of COVID-19 Primary Outcomes Between the Matched MDPCP Group and Nonparticipating Group, for Unknown Race and Ethnicity Beneficiaries Only

	**MDPCP Group (n = 2,536)**	**Non-Participating Group (n = 454)**	
**Measures**	**n (%)**	**n (%)**	**p-value**
**Vaccine Status**			.04
NOT vaccinated	188 (7.41)	50 (11.01)	
fully vaccinated with booster	1,909 (75.28)	318 (70.04)	
fully vaccinated without booster	368 (14.51)	72 (15.86)	
partially vaccinated	71 (2.8)	14 (3.08)	
**Booster Vaccine Status**			.02
No	627 (24.72)	136 (29.96)	
Yes	1,909 (75.28)	318 (70.04)	
**COVID-19 positive beneficiaries with monoclonal antibody infusion**	13 (10)	<11 (47.82)	.11
**COVID-19 positive beneficiaries with telehealth service claims**	165 (67.69)	18 (43.48)	.06

© 2023 Gruber E et al. *JAMA Network Open*.

eTable 17. COVID-19 Secondary Outcomes in the Matched MDPCP Group and Nonparticipating Group, for Unknown Race and Ethnicity Beneficiaries Only

	**MDPCP Group (n = 2,536)**	**Non-Participating Group (n = 454)**	
**Measures**	**n (%)**	**n (%)**	**p-value**
COVID-19 Positive Beneficiaries	130 (5.13)	23 (5.07)	.96
Beneficiaries with COVID-19 inpatient claims	17 (0.67)	<11 (<2.42)	.98
Beneficiaries with COVID-19 emergency department claims	11 (0.43)	<11 (<2.42)	.51
COVID-19 Death Count	<11 (<0.43)	0 (0)	.46
Average COVID-19 inpatient admission length of stay	9.26	9.33	.99

© 2023 Gruber E et al. *JAMA Network Open*.

eTable 18. Demographic and Risk Characteristic Comparison Between the Matched MDPCP Group and Nonparticipating Group, for White Beneficiaries Only

	MDPCP Group (n = 163,194)	Non-Participating Group (n = 29,161)	
**Measures**	**n (%)**	**n (%)**	**p-value**
**Age Category**			>.99
64 and Younger	8,436 (5.17)	1,503 (5.15)	
65 to 69	17,657 (10.82)	3,156 (10.82)	
70 to 74	42,404 (25.98)	7,579 (25.99)	
75 to 79	38,817 (23.79)	6,940 (23.8)	
80 to 84	26,527 (16.25)	4,738 (16.25)	
85 and Older	29,353 (17.99)	5,245 (17.99)	
**Gender**			.97
Female	95,234 (58.36)	17,014 (58.35)	
Male	67,960 (41.64)	12,147 (41.65)	
**Dual Medicare-Medicaid Eligibility Flag**			.96
No	153,306 (93.94)	27,392 (93.93)	
Yes	9,888 (6.06)	1,769 (6.07)	
**COVID-19 Vulnerability Index Score**			>.99
1 or 2	25,214 (15.45)	4,502 (15.44)	
3	50,841 (31.15)	9,080 (31.14)	
4	51,879 (31.79)	9,274 (31.8)	
5	35,260 (21.61)	6,305 (21.62)	
**County**			
Anne Arundel	18,465 (11.31)	3,297 (11.31)	
Baltimore	27,030 (16.56)	4,828 (16.56)	
Baltimore City	7,310 (4.48)	1,306 (4.48)	
Calvert	4,343 (2.66)	776 (2.66)	
Carroll	9,398 (5.76)	1,680 (5.76)	
Charles	2,765 (1.69)	493 (1.69)	
Frederick	10,808 (6.62)	1,932 (6.63)	
Harford	11,231 (6.88)	2,006 (6.88)	
Howard	9,843 (6.03)	1,759 (6.03)	
Montgomery	18,624 (11.41)	3,325 (11.4)	
Other	18,399 (11.27)	3,295 (11.3)	
Prince Georges	5,097 (3.12)	911 (3.12)	

© 2023 Gruber E et al. *JAMA Network Open*.

Saint Marys	4,646 (2.85)	830 (2.85)	
Washington	7,671 (4.7)	1,369 (4.69)	
Wicomico	3,907 (2.39)	700 (2.4)	
Worcester	3,657 (2.24)	654 (2.24)	
**Average HCC Score**			<.001
	1.3383	1.3719	
**Average ADI Score**			<.001
	29.18	28.59	

© 2023 Gruber E et al. *JAMA Network Open*.

eTable 19. Comparison of Uptake of COVID-19 Primary Outcomes Between the Matched MDPCP Group and Nonparticipating Group, for White Race Beneficiaries Only

	MDPCP Group (n = 163,194)	Non-Participating Group (n = 29,161)	
**Measures**	**n (%)**	**n (%)**	**p-value**
**Vaccine Status**			<.001
NOT vaccinated	21,288 (13.04)	5,417 (18.58)	
fully vaccinated with booster	107,778 (66.04)	17,139 (58.77)	
fully vaccinated without booster	30,479 (18.68)	5,662 (19.42)	
partially vaccinated	3,649 (2.24)	943 (3.23)	
**Booster Vaccine Status**			<.001
No	55,416 (33.96)	12,022 (41.23)	
Yes	107,778 (66.04)	17,139 (58.77)	
**COVID-19 positive beneficiaries with monoclonal antibody infusion**	974 (9.52)	144 (7.13)	<.001
**COVID-19 positive beneficiaries with telehealth service claims**	15,691 (61.76)	2,864 (54.06)	<.001

© 2023 Gruber E et al. *JAMA Network Open*.

eTable 20. COVID-19 Secondary Outcomes in the Matched MDPCP Group and Nonparticipating Group, for White Race Beneficiaries Only

	MDPCP Group (n = 163,194)	Non-Participating Group (n = 29,161)	
Measures	n (%)	n (%)	p-value
COVID-19 Positive Beneficiaries	10,235 (6.27)	2,020 (6.93)	<.001
Beneficiaries with COVID-19 inpatient claims	2,638 (1.62)	554 (1.9)	<.001
Beneficiaries with COVID-19 emergency department claims	1,480 (0.91)	305 (1.05)	.02
COVID-19 Death Count	844 (0.52)	220 (0.75)	<.001
Average COVID-19 inpatient admission length of stay	9.45	9.69	.51

© 2023 Gruber E et al. *JAMA Network Open*.
