# Supplementary material for: Association of Participation in the Maryland Primary Care Program With COVID-19 Outcomes Among Medicare Beneficiaries
Source: JAMA Netw Open. 2023 Jan 6;6(1):e2249791. doi: 10.1001/jamanetworkopen.2022.49791 (PMC9856987; doi:10.1001/jamanetworkopen.2022.49791)
Supplement: Supplement 2. — Data Sharing Statement [file jamanetwopen-e2249791-s002.pdf]

Data Sharing Statement

Gruber. Association of Participation in the Maryland Primary Care Program With COVID-19 Outcomes Among Medicare Beneficiaries. *JAMA Netw Open*. Published January 06, 2023. doi:10.1001/jamanetworkopen.2022.49791

Data
Data available: No

Additional Information
Explanation for why data not available: Data for this manuscript will not be shared. This data is owned and help by the Centers for Medicare & Medicaid Services (CMS) and is governed by a Data Use Agreement with the Maryland Department of Health, wherein the Maryland Department of Health has permission to share aggregate analyses only. Importantly, data is individual Medicare beneficiary claims data, and sharing this data would be a HIPAA violation.
